# Professional cleaning and risk of asthma – a Danish nationwide register-based study

**DOI:** 10.5271/sjweh.3997

**Published:** 2022-02-25

**Authors:** Camilla Sandal Sejbaek, Esben Meulengracht Flachs, Tanja Korfitsen Carøe, Harald William Meyer, Marie Frederiksen, Karen Bo Frydendall, Peder Wolkoff, Per Axel Clausen, Karin Sørig Hougaard, Vivi Schlünssen

**Affiliations:** 1 National Research Centre for the Working Environment, Copenhagen, Denmark; 2 Department of Occupational and Environmental Medicine, Bispebjerg and Frederiksberg University Hospital, Copenhagen, Denmark; 3 Department of Public Health, University of Copenhagen, Copenhagen, Denmark; 4 Danish Ramazzini Center, Department of Public Health – Environmental, Occupational and Health, Aarhus University, Aarhus, Denmark

**Keywords:** cleaner, cumulative exposure, Denmark, epidemiology, new-onset asthma, occupational asthma, occupational exposure

## Abstract

**Objective:**

This study aimed to investigate the risk of asthma among professional cleaners in a nationwide population-based study.

**Methods:**

Professional cleaners, aged 16–50 years, were identified according to the yearly assigned administrative job and industrial codes in a register-based, matched cohort study with other manual workers as references (1995–2016). Asthma was defined from national registers based on hospitalization and medication. Associations between recent and cumulative cleaning years and risk of asthma were estimated using Poisson regression, first in a full cohort and then in an inception cohort, among workers aged 16–20 years at the start of follow-up.

**Results:**

The risk of asthma was not increased for recent cleaning compared to references [adjusted incidence rate ratio (IRR_adj_) 1.02 [95% confidence interval (CI) 0.99–1.04]. Similar results were seen for the inception cohort, where cumulative years of cleaning were associated with increased risk of asthma, more prominent for the group with the maximum of six years of cleaning IRR_adj_ 2.53 (95% CI 1.38–4.64). Cumulative years of cleaning were associated with decreased risk of asthma, more pronounced for the maximum of ten compared to one year of cleaning [IRR_adj_ 0.74 (95% CI 0.63–0.88)].

**Conclusions:**

Asthma risk was increased in the inception cohort for cumulative years of cleaning but decreased in the full cohort. We could not confirm that recent work within cleaning was associated with increased risk of asthma. This may be due to healthy worker bias. Thus, we cannot rule out that long-term professional cleaning may be associated with increased risk of asthma.

More than 20 years ago, cleaning work was identified as a risk factor for prevalent asthma in a large population-based study (1) and some years later also for incident asthma in the same cohort (2). Within the last decades, several other studies have contributed evidence on the association between cleaning and the risk of asthma (3–9). The reviews mainly include cross-sectional studies on professional and household cleaning and the use of cleaning products from different sources and include human observational studies, exposure studies, and experimental mechanistic studies (3–7).

Being a professional cleaner has been associated with work-related asthma, especially chronic irritant induced asthma (10) and exacerbation of asthma (5). Specifically, agents like bleach (hypochlorite), ammonia, disinfectants, and mixing of products (eg, hypochlorite and hydrochloric acid) may cause asthma or worsen an existing asthma (5, 10). For professional cleaners, the risk for asthma, asthma symptoms, and impaired respiration seems to be dose-dependently associated to the use of cleaning spray and duration of cleaning (11–13). The increased risk of asthma is seen both for professional cleaners at workplaces and in private homes (3).

Ghost and colleagues (14) showed that adult onset asthma was associated with ever working in one of 18 occupations, among others cleaning jobs and jobs where exposure to cleaning agents are likely. In Denmark, only one population-based study has investigated asthma among professional cleaners. It showed that female cleaners had an increased risk of current asthma compared to women in administrative jobs [prevalence ratio 2.17 (95% confidence interval (CI) 1.47–3.21)] compared to administrators (15).

In the present study, we investigated for the first time whether professional cleaning was associated with the risk of asthma in a nationwide register-based, matched cohort study. We hypothesized that professional cleaners would have a higher risk of developing asthma compared to other manual workers and that the risk would increase with years of working in cleaning.

## Methods

### Study population

**Figure 1:**
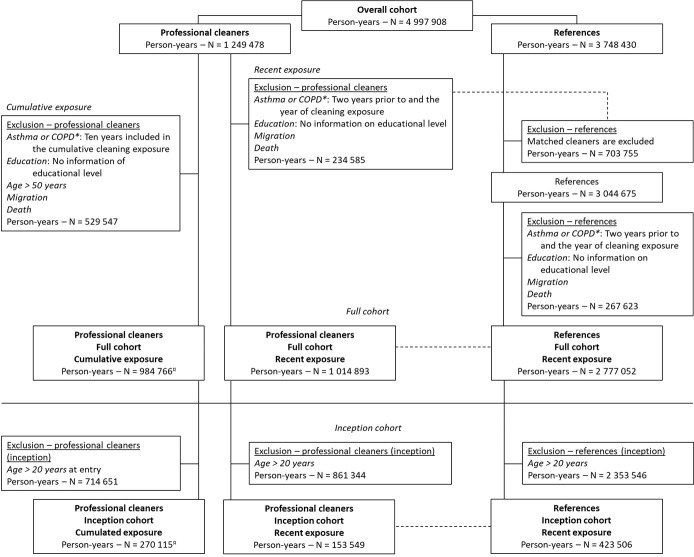
Flowchart for cleaners and references included in the analyses of recent and cumulative exposure in the full and in the inception cohort, respectively. *COPD=chronic obstructuve pulmonary disease; ¤ The person-years reflect the tital person-years included in the analyses. The cohort is expanded yearly with new individuals who have worked in cleaning for at least one year.

The study was conducted in the Danish Occupational Cohort, DOC*X, a national register-based workforce database (16). The database combines a number of health and administrative registers for all individuals in Denmark, who have been active at the Danish Labour market between 1976 and 2015 for at least one year (17). On a yearly basis, individuals are categorized according to the job held for the longest time period of that year. We included individuals aged 16–50 years who worked at least one year with professional cleaning activities (hereafter called cleaners) starting 1 January 1998, until 31 December 2015. For each included cleaner, we selected three workers in other manual or low skilled jobs as references (figure 1).

### Outcome

We retrieved information on asthma from two nationwide registers and categorized asthma according to the definitions given by Liu and colleagues (18): (i) The Danish National Patient Register (1994–2018) provides information on inpatient, outpatient, and emergency visits. An individual was considered diagnosed with asthma by having a hospital contact with International Classification of Diseases 10 (ICD-10) codes J45 (Asthma) or J46 (Status asthmaticus); (ii) From the Danish National Prescription Registry (1997–2018), we included information about asthma medication. An individual was considered diagnosed with asthma if the person redeemed a prescription at least twice within 12 months. The following medication codes of the Anatomical Therapeutic Chemical Classification System (ATC) were included: β2-agonists (R03AC02-04, R03AC12, and R03AC13), corticosteroids/inhaled glucocorticoids (R03BA01, R03BA02, and R03BA05), combination-products/fixed-dose combination of inhaled β2-agonists and glucocorticoids (R03AK06 and R03AK07), leukotriene receptor antagonists (R03DC03), and anti-IgE treatment (R03DX05).

Asthma was defined as at least one hospital contact due to asthma within the last 12 months and/or redemption of a prescription of one of the asthma medications at least twice within the past 12 months. The information from both registers was used to form a dichotomous outcome for asthma (yes/no).

### Exposure to cleaning

Cleaners were identified from their individual job titles based on the Danish International Standard Classification of Occupation (DISCO-88) or the Danish Industrial Classification of All Economic Activities 2007 [Dansk Branchekode (Danish Industrial Code) 2007 (DB07)].

We utilized the following four-digit DISCO-88 codes to define cleaners: 9131 (domestic helpers and cleaners) and 9142 (vehicle, window, and related cleaners). Individuals working in the mixed group 9132 (helpers and cleaners in offices, hotels and other establishments) were only included if they were also registered under the DB07 codes 8121 (general cleaning of buildings) or 8129 (other cleaning activities), to increase specificity. Finally, we included individuals that lacked a DISCO-88 code but had the industrial code 8121 (general cleaning of buildings) in order to include individuals working in companies with <10 employees for which reporting of DISCO-88 code is not mandatory in Denmark.

**Figure 2:**
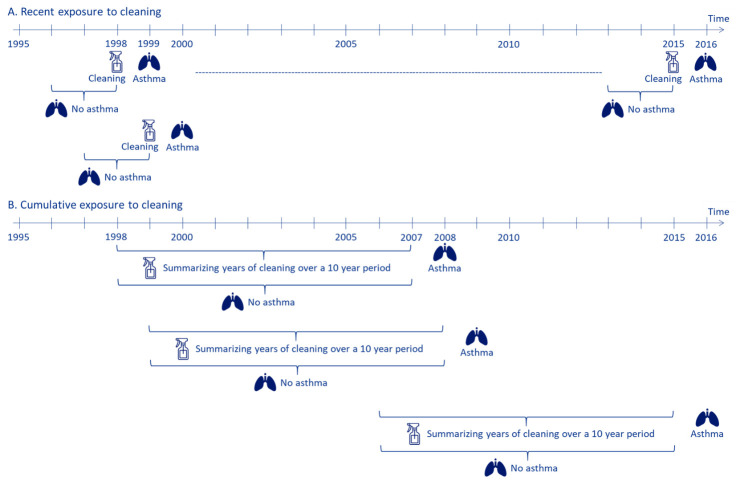
Study design of the analyses: (A) recent and (B) cumulative cleaning exposure in the full and in the inception cohort. (A) Cleaning = calendar year of exposure to cleaning work or other manual work. Asthma = risk of asthma. No asthma = a cleaner and a reference should be without asthma two years prior to and in the year of exposure. (B) Summarizing years of cleaning over a ten year period = year(s) of exposure to cleaning work (1–10 years). Asthma = risk of asthma. No asthma = a cleaner should be without asthma in the ten years of estimation of exposure to cleaning.

Exposure to cleaning was assessed in two ways (figure 2). Recent cleaning was assessed annually from 1998 to 2015. Participants were excluded if they had asthma or were diagnosed with chronic obstructive pulmonary disease (COPD) in any of the two years prior to or in the calendar year of exposure to cleaning (figure 1). Cumulative exposure to cleaning was assessed in a cohort with start of follow-up in year 2007 by summarizing the years of cleaning during the preceding 10 years (1998–2007 to 2006–2015). The participants were excluded if they were diagnosed with asthma or COPD in the ten years preceding the start of follow-up.

### References group

For recent exposure, each cleaner was annually matched on sex, age, and socio-economic position to three reference individuals. Matching on socio-economic position was conducted at the first-digit DISCO-88 code level and included the following job groups: five service workers and shop and market sales workers; seven craft and related trades workers; eight plant and machine operators and assemblers; and nine elementary occupations except the cleaners defined previously (figure 1). The references were solely included in the analyses investigating recent exposure (figure 2A). Due to the annual selection of references, each participant could contribute both with years as cleaner and years as reference, albeit not in the same year.

### Covariables

Information on COPD was retrieved from the Danish National Patient Register based on the following ICD-10 codes: J43, J43.0, J43.1, J43.2, J43.8, J43.9, J44, J44.0, J44.1, J44.8, or J44.9 (19). Highest obtained education level was obtained from the Education Register and grouped into five categories: primary school, upper secondary school, vocational training, bachelor’s programme, and master’s programme or higher. Furthermore, we included sex and age in the year of exposure (16–20, 21–30, 31–40, and 41–50 years).

### Analyses

We excluded participants with incomplete information on age, sex, highest obtained educational level, and workers residing outside Denmark during the period of interest (figure 1).

In the analysis of recent exposure, all matched references to the excluded cleaners were also excluded. Additionally, references, who independently had incomplete information, were excluded on an individual basis, meaning that a cleaner could have less than three references (figure 1). Cleaners were compared to their references by estimating the incidence rate ratios (IRR) of asthma (with a 95% CI) the year after exposure using Poisson regression models (figure 2A). Individuals were followed until the date of asthma or censored at the date of emigration, diagnosis of COPD, death, or the end of follow-up on 31 December each year. The crude analysis was adjusted for the year of cleaning, and the adjusted analysis were further adjusted for the highest obtained educational level, sex and age. The follow-up period was 1 January 1999 until 31 December 2016.

No external references were used in the analyses of cumulative cleaning; hence, all participants worked at least one year in cleaning (figure 1). Prior exposure to cleaning work was cumulated for the preceding ten years, starting in 2007. This procedure was repeated annually until 2015. Each year, the cohort was expanded with new individuals who had worked in cleaning for at least one year (figure 2B). The references consisted of individuals with one year of cleaning work. We used Poisson Regression models for estimating the IRR (95% CI) for asthma with start of follow-up on 1 January 2008. The individuals were followed until the date of asthma or censored at the date of emigration, diagnosis of COPD, death, or after ten years of follow-up, whichever came first. Follow-up was finalized on 31 December 2016. Crude and adjusted analyses were conducted as described for the analyses of recent exposure, including also the same co-variables.

### Sensitivity and subgroup analyses

To deal with potential healthy worker bias by ensuring complete exposure history and outcome information for the study participants, we defined an inception cohort of 16–20-year-old participants who had their first cleaning employment between 1998 and 2015. The analyses were similar to the analyses described above, for recent and cumulative exposure to cleaning, respectively (figures 2A and 2B). However, for cumulative exposure, a maximum of six years of cleaning could be accumulated over the course of ten years for this young cohort (20).

To investigate the impact of cleaning work among individuals without previous professional cleaning activities, we repeated the analysis of recent exposure in the subgroup of workers without employment in cleaning during the ten years prior to entrance into the cohort (2007–2015). Cleaners were compared with their matched references, also without cleaning in the past ten years.

## Results

In the analyses of recent exposure to cleaning, we included 360 479 cleaners and 1 218 692 references, contributing 1 014 893 and 2 777 052 person-years, respectively (table 1). The prevalence of asthma was similar (0.7%) for cleaner and reference years. About 2/3 were females. The oldest age group, 41–50 years, contributed the most person-years (33%), whereas the youngest age group (16–20 years) contributed the least (15%). Due to matching, the distribution of age was similar for cleaners and references. More than half of the cleaners had obtained primary education as their highest educational level, and 39% had obtained secondary education, whereas among the references 38% had obtained primary education, while more than half had a secondary education. The earlier years of the follow-up period contributed the most person-years ranging from 7.4% in 1998 to 3.4% in 2015.

**Table 1:** Characteristics of cleaners and references included in the analysis of recent exposure, contributing 3 767 830 person-years in total; and cleaners included in the cumulative exposure analysis contributing 978 873 person-years in total. Total number of unique individuals N=1 486 857.

	Recent exposure				10 years cumulative	
	
	Cleaners, person-years ^a^		References, person-years ^a^		Cleaners, total person-years	
		
	N	%	N	%	N	%
Individuals	360 479		1 218 692		176 297	
Total	1 014 893		2 777 052		984 766	
Risk time (years)	1 007 378		2 760 452		978 873	
Asthma						
Yes	7287	0.7	19 072	0.7	4900	0.5
No	1 007 606	99.3	2 757 980	99.3	979 866	99.5
Sex						
Female	695 681	68.5	1 901 112	68.5	624 714	63.4
Male	319 212	31.5	875 940	31.5	360 052	36.6
Age (years)						
16–20	153 549	15.1	423 506	15.3	133 706	13.6
21–30	257 906	25.4	690 985	24.9	355 120	36.1
31–40	274 042	27.0	754 577	27.2	219 727	22.3
41–50	329 396	32.5	907 984	32.7	276 213	28.0
Highest education						
Primary school	556 309	54.8	1 061 402	38.2	443 938	45.1
Upper secondary	394 999	38.9	1 560 322	56.2	438 780	44.6
Vocational training	21 506	2.1	70 177	2.5	24 094	2.4
Bachelors	32 809	3.2	72 096	2.6	59 533	6.0
Masters or higher	9270	0.9	13 055	0.5	18 421	1.9
Working year						
1998	75 045	7.4	205 771	7.4		
1999	71 459	7.0	197 552	7.1		
2000	64 067	6.3	177 353	6.4		
2001	64 072	6.3	177 479	6.4		
2002	60 420	6.0	167 219	6.0		
2003	57 339	5.6	159 021	5.7		
2004	59 848	5.9	165 855	6.0		
2005	60 864	6.0	168 402	6.1		
2006	47 536	4.7	131 550	4.7		
2007	62 873	6.2	170 336	6.1	54 748	5.6
2008	65 700	6.5	176 178	6.3	78 734	8.0
2009	59 373	5.9	160 235	5.8	92 774	9.4
2010	53 031	5.2	143 686	5.2	107 306	10.9
2011	49 687	4.9	134 711	4.9	116 538	11.8
2012	47 063	4.6	127 499	4.6	124 926	12.7
2013	44 888	4.4	121 392	4.4	131 939	13.4
2014	36 273	3.6	97 832	3.5	136 399	13.9
2015	35 355	3.5	94 981	3.4	141 402	14.4
Cleaning years, sum over 10 years						
1					357 600	36.3
2					218 547	22.2
3					123 275	12.5
4					78 268	7.9
5					53 327	5.4
6					40 238	4.1
7					30 934	3.1
8					26 266	2.7
9					25 058	2.5
10					31 253	3.2

a There is an overlap between cleaners and references; however, for one year an individual is only included as either cleaner or reference based on exposure to cleaning.

The population of cleaners included in the analysis of cumulative cleaning contributed 984 766 person-years. The distribution of asthma and sex was similar to the recent exposure cohort, apart from a larger proportion of workers aged 21–30 years. Due to the design of the study, the study population gradually increased in size until 2015. Among cleaners in the cumulative exposure population, 34% had worked only one year in cleaning, whereas 3% had ten consecutive years of cleaning.

**Figure 3:**
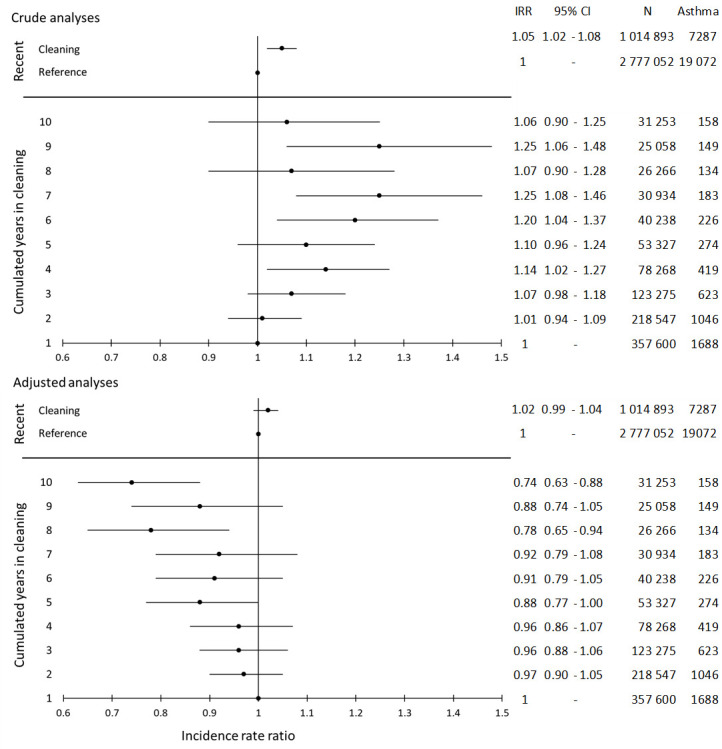
Crude and adjusted incidence rate ratio (IRR) and 95% confidence interval (CI) of work in cleaning, 1998–2015, and risk of asthma the following year in the full cohort, 3 767 630 person-years. Crude and adjusted IRR and 95% CI of the 10 years of cumulative exposure to cleaning, 2007–2015, and risk of asthma the following year, 984 766 person-years. [Crude analysis: adjustment for year of exposure. Adjusted analysis: Year of exposure + sex, age, highest level of education obtained.]

**Figure 4:**
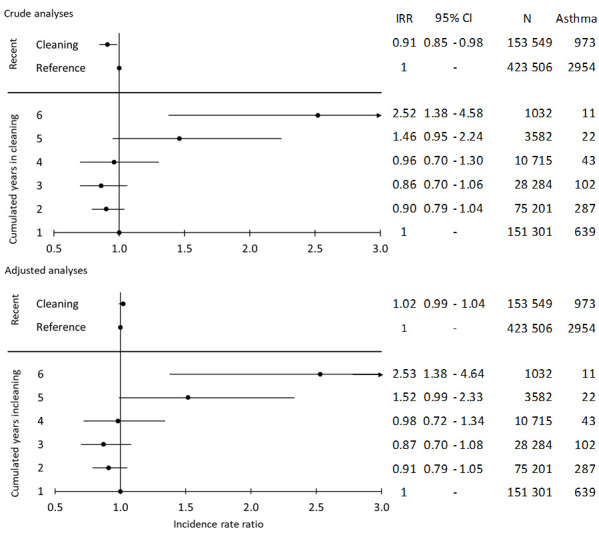
Crude and adjusted incidence rate ratio (IRR) and 95% confidence interval (CI) of work in cleaning, 1998-2015, and risk of asthma the following year in the inception cohort, 572 688 person-years. Crude and adjusted IRR and 95% CI of the ten year cumulative exposure to cleaning, 2007–2015, and the risk of asthma the following year, 270 115 person-years. [Crude analysis: adjustment for year of exposure. Adjusted analysis: Year of exposure + sex, age, highest level of education obtained.]

In the crude analyses, recent exposure to cleaning was associated with a slightly increased risk of asthma compared to the references. This attenuated in the adjusted analysis [adjusted IRR (IRR_adj_) 1.02 (95% CI 0.99–1.04), figure 3]. This was confirmed in the inception cohort [IRR_adj_ 1.02 (95% CI 0.99–1.04), figure 4]. The risk of asthma was also similar in the subgroup with no cleaning in the past ten years, IRR_adj_ 1.01 (95% CI 0.91–1.12; data not shown). This subgroup comprised 108 732 person-years among cleaners and 281 760 person-years among references.

The analysis of cumulative years of cleaning during the preceding ten years indicated an increased risk of asthma in the crude analysis with the highest risks for seven and nine years of cleaning, IRR 1.25 (95% CI 1.08–1.46) and IRR 1.25 ( 95% CI 1.06–1.46), respectively. However, in the adjusted analysis, the risk of asthma decreased with increasing number of years in cleaning, most pronounced for eight or ten years [IRR_adj_ 0.78 (95% CI 0.65–0.94) and IRR_adj_ 0.74 (95% CI 0.63-0.88), respectively, figure 3].

In the inception cohort, the results for cumulative exposure is only presented up to six years of work in cleaning due to small numbers of asthma events (N=270 115 person-years, asthma events=1104). Both unadjusted and adjusted analyses showed higher risks of asthma with increasing number of cleaning years. IRR_adj_ for five and six years of cleaning were 1.52 (95% CI 0.99–2.33) and 2.53 (95% CI 1.38–4.64), respectively (figure 4).

## Discussion

The overall aim of the study was to investigate whether professional cleaning increases the risk of asthma. The risk of asthma was increased in a dose-dependent manner in the inception cohort, where the cleaners were 16–20 years of age at inclusion but not in the full cohort with many prevalent hires, where we observed a decreased asthma risk with increased cleaning years. This indicates that a healthy worker bias may occur among cleaning professionals. We could not confirm that recent professional cleaning, ie, cleaning within the previous year, was associated with an increased risk of asthma.

To account for the selection processes related to duration of exposure before inclusion in the present study, we used an inception cohort where we assume prior exposure would be negligible. The findings of higher risks of asthma with increasing number of cleaning years from the inception cohort are partly supported by previous findings conducted in populations of cleaners in general [summarized in Folletti et al (3)], based on two studies. The study by de Fatima Macaira et al (21) showed association between duration of exposure to cleaning and work-related asthma, while Obadia et al (22) did not confirm these results.

Most published epidemiological studies indicate an effect of cleaning activities and cleaning/disinfecting agents on prevalence or incidence of asthma (23). This was, however, not confirmed by our analyses of recent cleaning. Job title constitutes a crude measure of cleaning without details on type or level of exposure to cleaning agents. We can therefore not conclude that recent cleaning is not related to incident asthma, but in light of our results, working in cleaning does not seem to have a large impact on incident asthma at the population level.

We interpret the inverse association between cumulative years with cleaning and asthma to be primarily an effect of healthy worker bias, which is a selection process. It can be described by (i) workers who are less fit for exposure during their employment are more likely to leave this work early and thereby accumulate less exposure; and (ii) a confounding effect, which relates to differences in health-related risk factors in workers with frequent job change and workers, who are established at the labor marked (19, 24, 25). This is supported by the findings in a follow-up study by Dumas and colleagues (26). They did not find associations in standard analyses of airborne exposure and asthma (including industrial cleaning agents) but observed clear indications of positive associations between exposure to allergens/irritants and asthma using a marginal structural model as a way to take the healthy workers bias into account.

In the review by Vincent et al (7), most studies reported increased risk of self-reported, physician-diagnosed asthma following work in cleaning. The present study also used physician-diagnosed asthma but based on either a physician’s diagnosis upon hospital contact or redeemed prescriptions prescribed by a physician. The discrepancy between our finding on recent exposure and some previous findings could be due to differences in the information on exposure and outcome, as most previous studies relied on self-reported questionnaire data on exposure and outcome (26).

Furthermore, the composition of reference groups can be of significance to the results of our study. Asthma occurs more frequently among individuals with low socioeconomic position (27). According to a review by Jaakkola & Jaakkola (28) cleaners have increased risk of developing asthma compared to reference groups of administrative and professional employees. As socio-economic position and related lifestyle factors are associated with the risk of developing asthma (12), it can be difficult to select an ideal comparison group in studies investigating occupational exposures in cleaners (27). We matched our cleaners to references with similar socio-economic position via job titles but still found the references to be somewhat higher educated (table 1). Our results showed that the highest obtained education was the most influencing factor on the results for of recent cleaning references (data not shown). On the other hand, job titles at a similar socio-economic position as cleaners might carry their own asthma inducing exposures (eg, unskilled work in the food and industry and construction), leading to underestimation of the true risk attributable to cleaning work.

One main reason for studying work in cleaning and onset of asthma is the inherent potential for cleaning agents to cause respiratory disorders (29). For example, Weinmann and colleagues (30) reported a three-fold higher odds of a doctor-diagnosed asthma for individuals aged 20–24 years exposed to disinfectants. Most of the epidemiological studies included in the review by Clausen and colleagues (9) indicated an increased risk of asthma due to use of cleaning agents in spray form both among professional and non-professional cleaners. Unfortunately, it is not possible to retrieve information on the type and use of cleaning agents in this cohort due to the register-based design of the study. Furthermore, very little is known on the asthma inducing potential of substances in spray cleaning products (31).

In the analysis of recent exposure in the full cohort, we did a post hoc analysis of individuals with asthma. The data showed that the risk of asthma increased over time. Furthermore, the data also showed that asthma events consisted of both new-onset and re-occurring asthma (data not shown). For earlier asthmatics to be included in our analyses, their prior asthma had to have occurred more than two years prior to the year of exposure to cleaning due to the exclusion criteria. The risk of re-occurring with asthma increased over time from 6% of the cohort in 1999 to 40% in 2014 (data not shown). However, the subgroup analyses of recent exposure in cleaners without asthma in the past ten years showed similar findings as for the original analyses indicating that inclusion of re-occurring asthma probably did not affect our results in a large degree.

In a post hoc analysis, we showed that 7964 cleaners (corresponding to 3.4% of the excluded cleaners; data not shown) were left out of the analysis due to onset of asthma within the year of exposure in cohort used in the analysis of recent exposure (full cohort). This could indicate that some cleaners will be at risk of early onset asthma within the year of exposure; however, the magnitude of this problem is not possible to estimate in our study, because of the register-based design. The total proportion of excluded cleaners left out of the analysis due to asthma two years prior to and in the year of exposure was 21.9%.

The marked change from excess risk to protective effect in the crude and adjusted analyses of cumulative cleaning, respectively, occur due to adjustment for age, whereas sex and education had no or small influence on the associations. As age and cumulated years of cleaning are somewhat correlated, the adjustment for age might over-adjust, and thus, attenuate excess risk. As we do not, however, find the same degree of change due to adjustment for age for the associations in the inception cohort, we anticipate over-adjustment to be a minor problem.

### Strengths and limitations

Due to the nationwide, register-based design, it was possible to include a large population of cleaners over an extensive time period; hence, we included more than one million person-years of cleaning. The references were chosen based on similarity to the cleaners’ socio-economic position. Group 6 “skilled agricultural and fishery workers”, the one-digit DISCO-88 code level, was, however, omitted from the references since these job groups carry an increased risk of asthma (32). All data was collected prospectively in nationwide registers and analyzed as such. Due to the register-based design, the information on exposure and outcome was collected independently of each other. Exposure status was, furthermore, assessed based on job titles supplied by the employer. This minimizes the risk of reporting bias for exposure, albeit at the expense of exposure accuracy. Our data enabled us to investigate both the recent and the long-term effects of cleaning.

Both full- or part-time work, sickness absence, and maternity leave may have influenced the findings, but this information was, unfortunately, not available. We only included individuals if their main occupation was cleaning, but – by disregarding part-time work and absence from work – we overestimated the exposure, and thereby potentially underestimated the association between cleaning exposure and asthma.

Work in cleaning is associated with a poorer health status due to competing risks (eg, musculoskeletal disorders) and low socioeconomic status. By using other manual workers as the reference group, we believe, we partly overcame the difference in competing risks between cleaners and references in the analyses of recent exposure. In the analyses on cumulated exposure, we compare cleaners to cleaners, and we do not believe differences in competing risks within the cleaning group is a substantial problem.

We did not have information on lifestyle factors, most importantly smoking, a recognized risk factor for asthma. We cannot rule out smoking confounded our results, and we cannot foresee the direction of a possible bias, despite our reference group is assumed to have similar smoking habits as the cleaners. In the DOC*X cohort, exposure matrices for six different lifestyle factors was developed, including smoking (33). These matrices provide information based on the DISCO-88 codes; hence, everyone working in cleaning will be assigned the same level of, eg, smoking, and the matrices were therefore not useful in the current analysis.

Asthma was based on information from two nationwide registers, on hospital diagnoses and redemption of prescriptions of asthma medication. Therefore, we investigated asthma in general and not asthma registered as work-related asthma. Work-related asthma implies per se a causal association between work and asthma, and would therefore not have been useful in the current analyses.

The analyses were conducted as complete case analysis, as cleaners and references without information on highest obtained educational level were excluded from the cohort. The cleaning profession primarily consists of unskilled workers, and furthermore, an unknown proportion of the cleaners are immigrants for whom we do not have information about their educational level. Thus, the lack of information on educational level might lead to an underestimation of asthma risk.

### Concluding remarks

In conclusion, an increased asthma risk was found in the inception cohort for cumulative cleaning years but not in the full cohort with many prevalent hires, indicating that healthy worker bias was at play. In our study, recent work within cleaning was not associated with an increased risk of asthma compared to references of similar socio-economic status.

Even though the association between cleaning and asthma has previously been investigated in several studies, we could address under-investigated issues including age at exposure and the impact of cumulative years of exposure to cleaning. Future studies should make an effort to understand differences between newly hired and experienced cleaners. An important next step would be to combine the advantages of a prospective nationwide study with more detailed information about cleaning exposures, eg, specific agents or modes of application.
